# Global hotspots and trends of nutritional supplements for sick populations from 2000 to 2024

**DOI:** 10.3389/fnut.2025.1497207

**Published:** 2025-01-28

**Authors:** Chaofan Shi, Haitao Liu, Te Fu, Yuanquan Li, Haichang Zhao, Feiyue Liu

**Affiliations:** ^1^College of Physical Education, Henan University, Kaifeng, Henan, China; ^2^Research Center of Sports Reform and Development, Henan University, Kaifeng, Henan, China; ^3^Institute of Physical Fitness and Health, Henan University, Kaifeng, Henan, China

**Keywords:** sick people, nutritional supplements, malnutrition, bibliometrics, CiteSpace, VOSviewer

## Abstract

**Background:**

Nutritional supplements (NS) can help patients by providing various nutrients such as essential vitamins and minerals, helping to prevent and recover from diseases. This study provides a broad overview of the field of NS for sick people through bibliometrics and visualization analysis, to analyze the status and development dynamics, explore the popular research questions and directions, and reveal the development trends and research frontiers.

**Methods:**

We searched the Web of Science Core Collection databases for literature related to NS for diseased populations from 2000 to 2024. A total of 1,550 articles were included in the analysis after screening. Analyses performed using CiteSpace and VOSviewer software.

**Results:**

The field of NS for the sick population has witnessed an overall rapid growth in the number of publications, which is divided into three phases: 2000–2008 was the exploratory phase, 2009–2017 was the sustained development phase, and 2018 to date is in the rapid development phase. Research focuses on dietary supplementation, oxidative stress, *in vitro* injections, development, antioxidant activity, double-blind trials, lipid supplements, functional foods, the health of diseased populations, and the risks of NS.

**Conclusion:**

Different supplements each possess unique benefits and should be chosen according to the type of disease to ensure they contain the corresponding nutrients. Vitamin supplements are widely mentioned among patient populations across the globe. Future trends may focus on applying nutritional supplements in gut microbiota and bioactive compounds. Researchers frequently mention the application of NS in women, infants, and children. It should continue to be monitored and optimized in the future to enhance its therapeutic effects, thereby accelerating patients’ recovery and improving their quality of life.

## 1 Introduction

Nutritional supplements (NS) contain one or more natural products, including herbs, vitamins, minerals, probiotics, amino acids, and fatty acids, among other nutrients, and are important when a person’s nutritional status is not conducive to good health (1, 2). The presence of malnutrition in a sick population is detrimental both physiologically and clinically, impairing quality of life and delaying recovery from the disease (3), and data suggest that disease-related malnutrition doubles the risk of death in hospitalized patients and triples the mortality rate of older adult patients in the hospital and after discharge (4). Nutritional support can be given orally, as well as enteral nutrition (tube feeding) and parenteral nutrition (5). Oral NS (ONS) is used for specific medical purposes to increase energy and nutrient intake, which is economical, convenient, and readily accepted by patients (6). ONS are liquid foods which are also used to improve nutrient intake in older adults and patients with a variety of health and dietary problems. The importance of enteral nutrition and ONS as artificial means of nutritional support has been elaborated (7). In a variety of hospital and community-based patients, the use of ONS has been shown to improve energy and nutrient intake, increase body weight and functional outcomes, reduce mortality and complications, and shorten the length of stay in hospitalized patients when compared to routine clinical care (8). During radiotherapy, many individuals with esophageal cancer have difficulty consuming solid foods, and regular semi-liquid and liquid diets are unable to meet their target energy requirements (9). The use of ONS at this time can supplement energy and protein deficiencies in individuals with esophageal cancer, maintain and increase their weight, improve nutritional status, and enhance their quality of life (10). All these effects allow adequate nutritional intake in patients with insufficient food intake and reduce the complications associated with malnutrition (11).

A balanced diet is the primary source of the body’s vital nutritional needs, and nutritional deficiencies are usually associated with lower average daily body requirements, with consequent health problems; multivitamin and mineral (MVM) supplements help to fill in the gaps where the daily diet does not meet the body’s nutritional needs (12). In addition, these supplements help to prevent a wide range of diseases and are better tolerated without increasing the risk of death (13). The use of vitamin supplements helps to reduce the risk of myocardial infarction and cardiovascular disease in groups such as adults aged 45–75 years with no history of myocardial infarction and women aged 49–83 years with no history of cardiovascular disease (14). MVM in filling critical nutritional gaps has been found to prevent anemia, neural tube defects, and bone disease by providing enough folate, iron, vitamin B12, and vitamin D (15). In addition, it can also be beneficial in preventing cancer or delaying cataracts, as well as in cognitive performance (16). A supplement containing 120 mg of ascorbic acid, 30 mg of vitamin E, 6 mg of beta-carotene, 100 μg of selenium, and 20 mg of zinc reduced overall cancer incidence by 31% and mortality by 37% in men (45–60 years old) after being ingested consistently by patients for 7.5 years (17). In the study, high doses of 3 vitamins with antioxidant properties (500 mg/d vitamin C, 400 IU/d vitamin E, and 15 mg/d *β*-carotene) along with zinc (80 mg zinc oxide) significantly reduced the risk of ocular disease, lens clouding, and nuclear cataracts by 28, 16, and 25%, respectively (18, 19). An Australian randomized controlled trial (RCT) of supplementation that included about 50 vitamins, minerals, and herbs found that it improved contextual recognition memory in men aged 50–74 years (20). Nutritional supplementation can positively affect the incidence of postoperative complications, especially in malnourished patients (21). The risks of malnutrition include a compromised immune system, increased incidence and severity of infections, poor wound healing, higher frequency of minor and serious complications, longer recovery time, longer hospitalization, and higher mortality (22). Studies have shown that 40–55% of hospitalized patients are malnourished or at risk of malnutrition, and the risk of malnutrition increases with age. Malnourished surgical patients are 2–3 times more likely to have complications and increased mortality than the general population (23). Thus, NS is of great significance for disease prevention and patient rehabilitation.

Bibliometrics is the discipline that quantifies bibliographic materials by analyzing research areas and identifying major trends (24). It includes the analysis of research topics, countries, journals, and institutions (25–28). In this paper, we searched for studies on NS in sick populations from 2000 to 2024. The aim is to find out the research hotspots and explore the development trend in NS for sick people, to quickly provide high-value information and promote the rapid development of research in this field.

## 2 Data collection and research methods

### 2.1 Data collection

**Figure 1:**
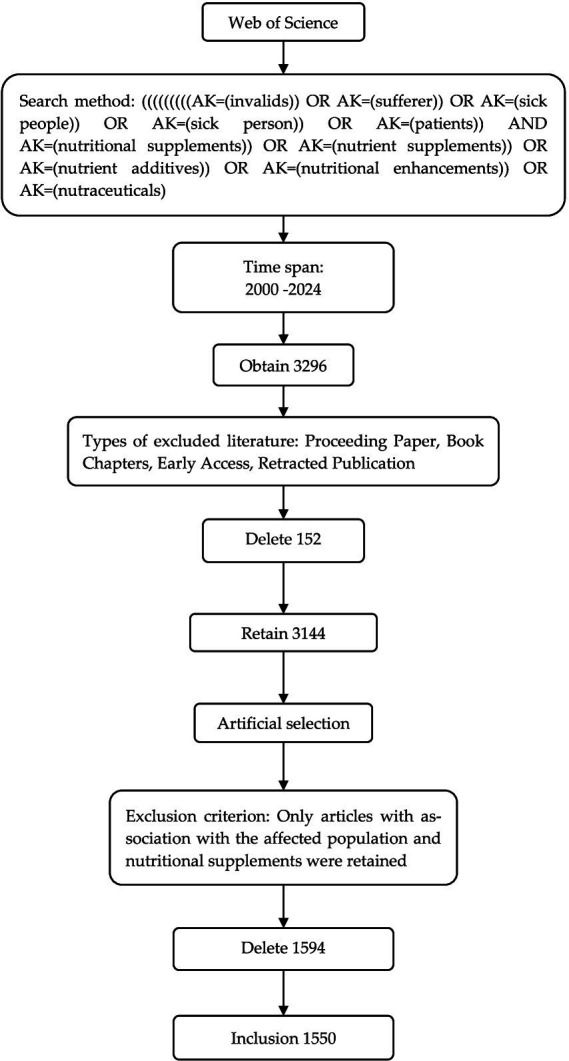
Flowchart of literature screening.

In this study, the Web of Science Core Collection (WOSCC) database was used as the data source, and the period was set from January 1, 2000, to April 21, 2024. This paper was searched using the Author Keyword (AK) form with the search format [AK = (invalids)] OR [AK = (sufferer)] OR [AK = (sick people)] OR [AK = (sick person)] OR [AK = (patients)] AND [AK = (nutritional supplements)] OR [AK = (nutrient supplements)] OR [AK = (nutrient additives)] OR [AK = (nutritional enhancements)] OR [AK = (nutraceuticals)] search. The total number of articles was 3,296, excluding Proceeding Papers, Book Chapters, Early Access, Retracted Publication. After excluding 152 articles, 3,144 articles were left and were manually screened. Only articles relevant to diseased populations and NS were retained. We further excluded 1,594 articles, and 1,550 articles were selected (Figure 1).

### 2.2 Research methods

In this study, Citespace was used to analyze, detect, and visualize trends and patterns in the scientific literature (29), and VOSviewer was used to construct and view bibliometric maps (30). Citespace was used to map knowledge, and VOSviewer was used to construct and view bibliometric maps. Institutions, authors, keywords, and countries of 1,550 screened documents were visualized and analyzed, and the trend of the number of publications and maps of author collaboration, institutional collaboration, geographic distribution, and keyword were drawn to highlight key nodes and research hotspots and to visualize the field of NS for diseased populations, respectively.

## 3 Results

### 3.1 Annual volume of communications

**Figure 2:**
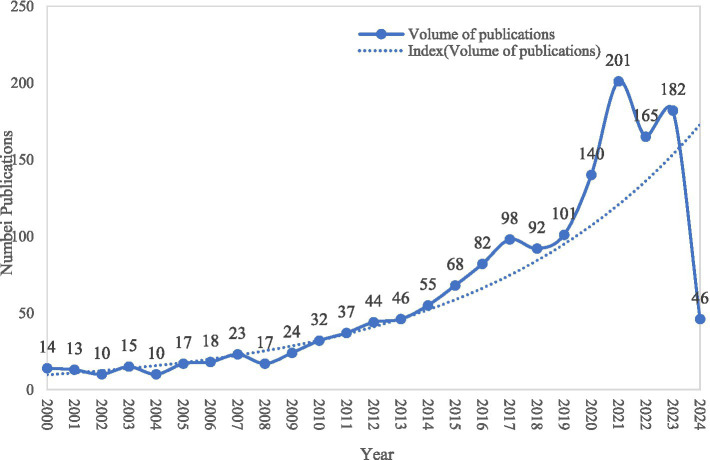
Trends in the number of publications per year. The solid blue line represents the annual variation in the number of publications, while the dashed blue line indicates the overall trend in publication volume.

A total of 1,550 relevant articles were published in this field from 2000 to 2024, and the overall trend of a sharp increase in the number of annual publications can be seen in Figure 2. The number of publications was relatively stable from 2000 to 2008, which increased per year from 2009 to 2017. There was a rapid increase in the number of publications after 2018 and up to 2023.

### 3.2 Journal analysis

The top 10 journals that issued articles are shown in Table 1. Research on NS for sick people primarily involves nutritional, clinical, and nursing journals. The top 10 journals had a total of 291 articles, accounting for 18.8% of the total number of articles, indicating that these journals are the mainstay of research in this field and are in the lead. *Nutrients*, with 64 articles and a citation frequency of 1,965, ranked at the top in terms of both articles and high citations, indicating that the journal is of high quality and is more popular in the field. The second-ranked journal is the *Journal of Nutrition*, which has 41 articles. In third and fourth places are the *American Journal of Clinical Nutrition* and *Maternal and Child Nutrition*, with 34 articles each. Five of the top 10 journals are from Switzerland.

**Table 1:** Top 10 journals by publications.

Rank	Periodical	Publications	Citations	Country	Category	IF (2023)	CiteScore (2023)
1	*Nutrients*	64	1965	Switzerland	Q1	5.8	9.2
2	*Journal of Nutrition*	41	886	United States	Q2	3.7	7.6
3	*American Journal of Clinical Nutrition*	34	1,318	United States	Q1	6.5	12.4
3	*Maternal and Child Nutrition*	34	541	England	Q3	2.8	7.7
4	*International Journal of Molecular Sciences*	26	971	Switzerland	Q1	4.9	8.1
5	*Molecules*	23	539	Switzerland	Q2	4.2	7.4
6	*Critical Reviews in Food Science and Nutrition*	19	906	United States	Q1	7.3	22.6
6	*Trends in Food Science and Technology*	19	997	England	Q1	15.1	32.5
7	*Foods*	16	215	Switzerland	Q1	4.7	7.4
8	*Frontiers in Nutrition*	15	79	Switzerland	Q2	4.0	5.2

### 3.3 Analysis of highly cited literature

Among the top 10 cited literature (Table 2), “Phytochemicals: nutraceuticals and human health” by Dillard, CJ is the most highly cited article with 706 citations, which was published in 2000.

**Table 2:** Top 10 most frequently cited literature.

Rank	Title	Author	Periodical	Publisher	Country	Citations	Year
1	Phytochemicals: nutraceuticals and human health (105)	Dillard, CJ	*Journal of the Science of Food and Agriculture*	Wiley	England	706	2000
2	Reactive oxygen species (ROS) and cancer: Role of antioxidative nutraceuticals (106)	Prasad, S	*Cancer Letters*	Elsevier Ireland Ltd.	Netherlands	621	2017
3	Regulation of survival, proliferation, invasion, angiogenesis, and metastasis of tumor cells through modulation of inflammatory pathways by nutraceuticals (107)	Gupta, SC	*Cancer and Metastasis Reviews*	Springer	United States	598	2010
4	Interactions of gut microbiota with functional food components and nutraceuticals (103)	Laparra, JM	*Pharmacological Research*	Academic Press Ltd.–Elsevier Science Ltd.	England	464	2010
5	Oxidative stress in cardiovascular diseases: still a therapeutic target? (108)	Senoner, T	*Nutrients*	MDPI	Switzerland	421	2019
6	Lipid-lowering nutraceuticals in clinical practice: position paper from an international lipid expert panel (109)	Cicero, AFG	*Nutrition Reviews*	Oxford Univ Press Inc.	United States	402	2017
7	A critical approach to evaluating clinical efficacy, adverse events and drug interactions of herbal remedies (110)	Izzo, AA	*Phytotherapy Research*	Wiley	England	376	2016
8	Neuroinflammation in the pathogenesis of Alzheimer’s disease. A rational framework for the search of novel therapeutic approaches (111)	Morales, I	*Frontiers in Cellular Neuroscience*	FRONTIERS MEDIA SA	Switzerland	371	2014
9	Milk protein-derived peptide inhibitors of angiotensin-I-converting enzyme (112)	FitzGerald, RJ	*British Journal of Nutrition*	Cambridge Univ Press	England	362	2000
10	Targeting inflammation-induced obesity and metabolic diseases by curcumin and other nutraceuticals (113)	Aggarwal, BB	*Annual Review of Nutrition*	Annual Reviews	United States	333	2010

### 3.4 Analysis of countries/regions

**Figure 3:**
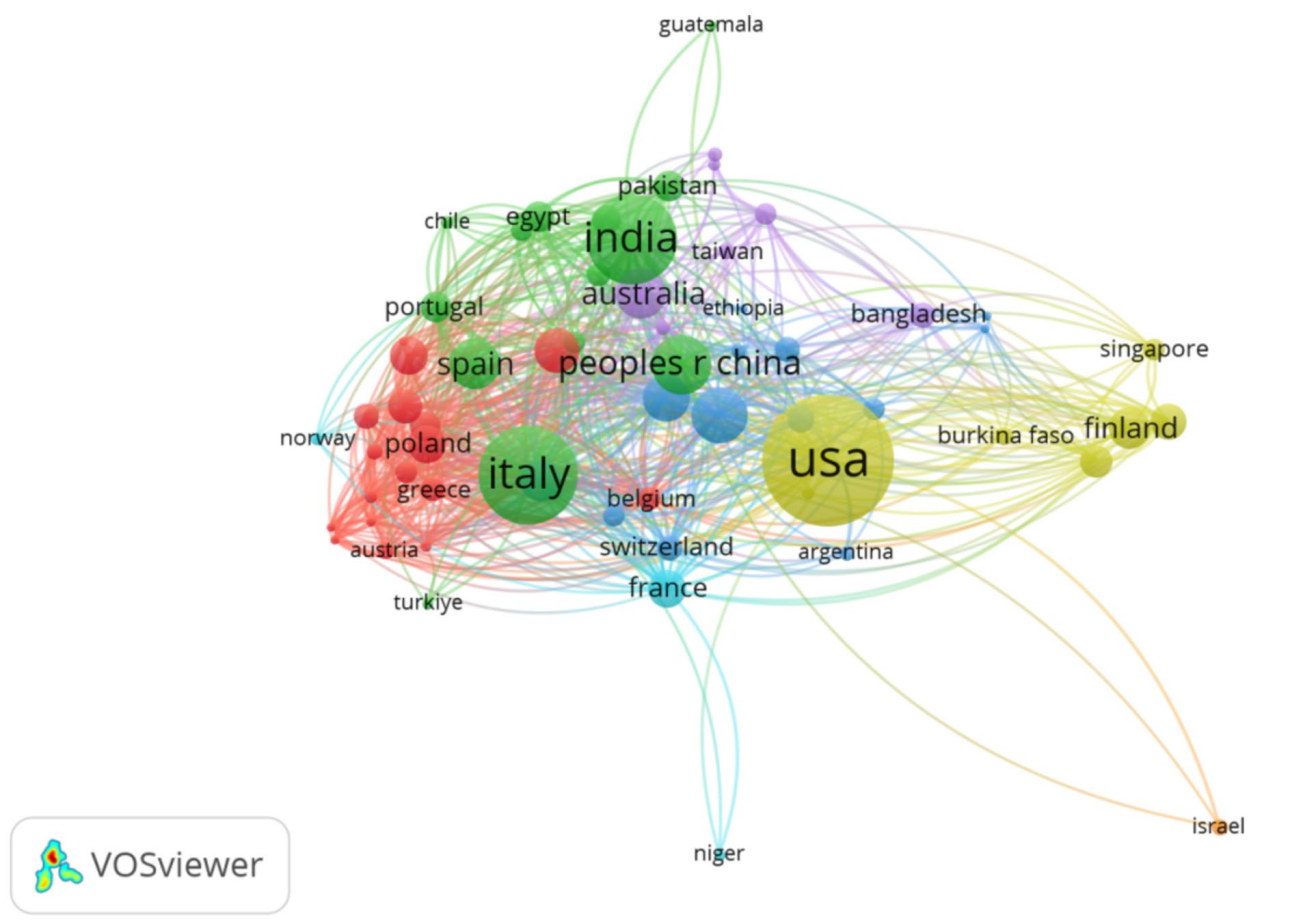
Distribution of publications by country region. The size of the nodes represents countries’ contributions, while the connecting lines between nodes indicate collaboration among nations. The thickness of the lines is positively correlated with the strength of the connection, and the color of the lines represents different clusters of countries engaging in spontaneous collaboration.

**Figure 4:**
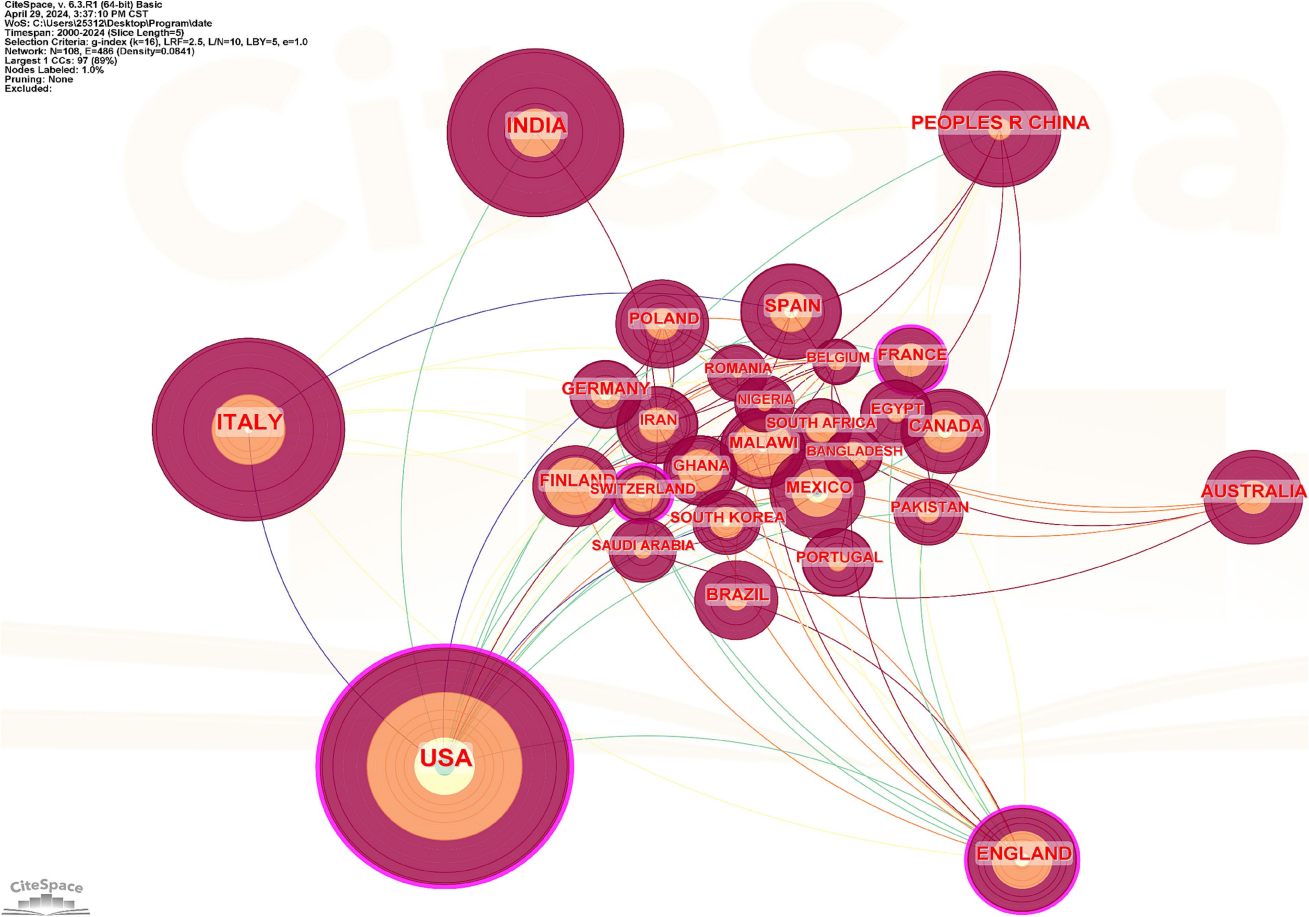
Distribution of national and regional distribution of publications. The size of the nodes is positively correlated with the number of articles published by each country. The lines represent the cooperative relationships between countries, and the number of lines positively correlates with the cooperation density.

Table 3 demonstrates that the United States is in 1st place in terms of the number of publications in this field, with a total of 406 publications, accounting for 26.2% of the approximate total. Italy ranks 2nd with 255 articles, accounting for about 16.4% of the total number of articles. India ranked 3rd with 217 articles, accounting for about 14% of the total. China ranked 4th with 107 articles, accounting for 6.9% of the total number of articles. The distribution charts (Figures 3, 4) show the cooperation relationship and density of each country. The size of the nodes in Figure 3 represents countries’ contribution, while the connecting lines between nodes indicate collaboration among nations. The thickness of the lines is positively correlated with the strength of the connection, and the color of the lines represents different clusters of countries engaging in spontaneous collaboration (31, 32). As shown in Figure 4, there are 108 nodes and 512 links; the network density is 0.0886. The size of the nodes is positively correlated with the number of articles published by the country. The lines represent the cooperative relationships between countries, and the number of lines positively correlates with the cooperation density (33, 34). Grid density evaluates the tightness of connections in a knowledge graph based on links and nodes. Indicating more cooperation in this field, close cooperation has been carried out mainly centering on the United States, Italy, India. Centrality is an important indicator for determining the evolution process of a discipline and predicting its development trends. The level of centrality represents the degree of importance to the development of the discipline (35). The centrality index of each country shows that some countries are at the top of the field in terms of the number of publications but have a centrality of 0, such as Mexico, Malawi, and Ghana. This indicates that the field is a hot topic of research within these countries, but there is no communication and cooperation with the outside world yet.

**Table 3:** Top 10 countries/regions in terms of number of publications.

Rank	Country	Publications	Percentage (%)
1	United States	406	26.2
2	Italy	255	16.4
3	India	217	14.0
4	China	107	6.9
5	England	94	6.1
6	Spain	79	5.1
7	Australia	78	5.6
8	Canada	69	5.0
9	Brazil	64	4.1
10	Finland	59	3.8

### 3.5 Analysis of institutional and author collaboration

**Figure 5:**
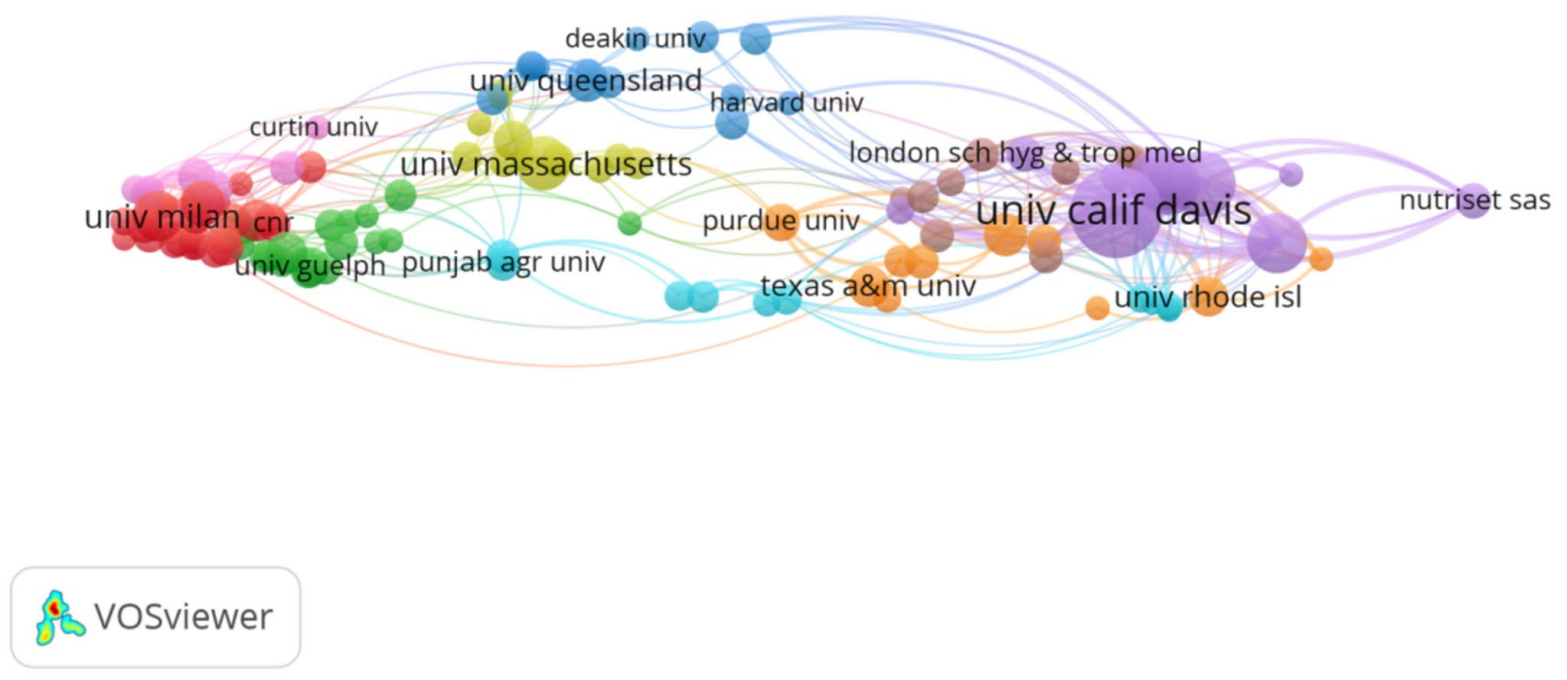
Collaborative mapping of research institutions. The size of the nodes is proportional to the publication volume of the institutions, while the thickness of the connecting lines indicates the strength of collaboration between the institutions. Different color clustering reflects the cooperative relationship between institutions.

**Figure 6:**
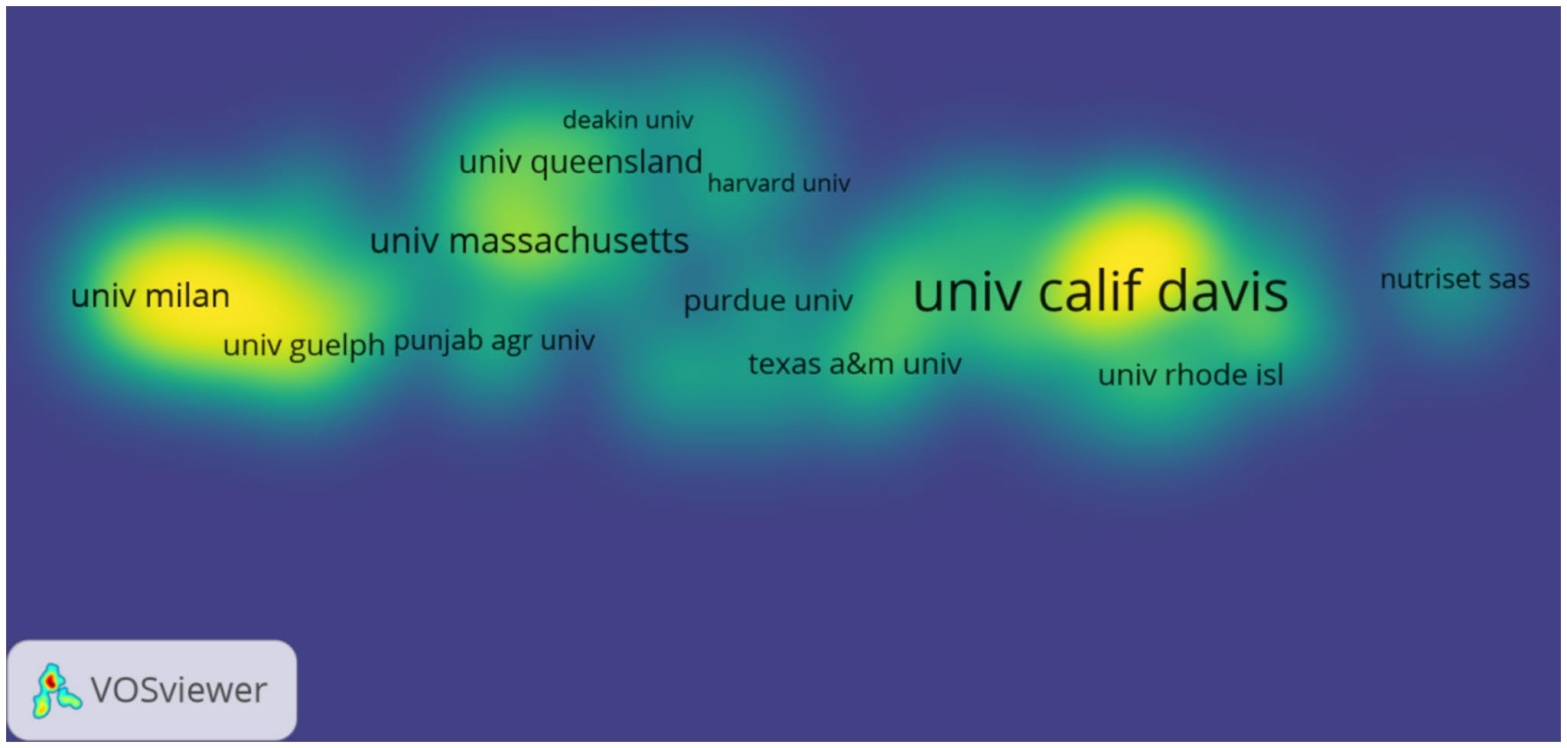
Density of cooperation among research institutions. The density of institutional cooperation is positively correlated with color expression: the higher the density, the more the color tends toward yellow and intensifies; when the density decreases, the color shifts toward the blue range.

Figures 5, 6 show the partnership and collaboration density between institutions. Universities dominate the volume of institutional publications. The University of California (UC) System tops the list with 102 publications. The UC Davis is 2nd with 89 articles. Other influential institutions are Tampere University, Tampere University Hospital, University of Malawi, University of Ghana, Council of Scientific and Industrial Research (CSIR) – India, Egyptian Knowledge Bank (EKB), Indian Council of Agricultural Research (ICAR), and University of Massachusetts System. Among the top 10 institutions in terms of the number of publications, three are from the United States and two each from India and Finland (Table 4). This indicates that these countries emphasize the development of research in this field, and a greater demand for research results in this field may exist due to the large number of sick or chronically ill people in their countries.

**Table 4:** Top 10 institutional issuances.

Rank	Country	Institution	Publications
1	United States	UC System	102
2	United States	UC Davis	89
3	Finland	Tampere University	56
4	Finland	Tampere University Hospital	48
5	Malawi	University of Malawi	38
6	Ghana	University of Ghana	35
7	India	CSIR-India	33
8	Egypt	EKB	31
8	India	ICAR	31
9	United States	University of Massachusetts System	29

**Figure 7:**
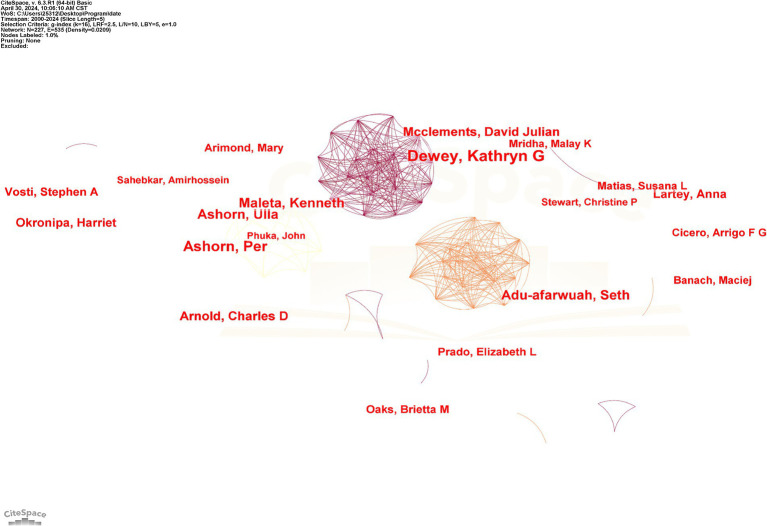
Mapping of author collaboration networks. Each node signifies an author, while each line represents a collaborative partnership between two authors. The more lines there are, the closer the collaboration. The size of the author’s name is directly proportional to the number of their publications.

In the author collaboration network mapping (Figure 7), the number of nodes and connecting lines, size, and co-occurrence frequency indicate the relationship and strength of collaboration between authors (36). In Figure 7, there are 227 nodes and 535 connecting lines, and the network density is 0.0209. Figure 7 shows that Dewey, Kathryn G., and Ashorn, Per are prolific authors in the field with high impact. As shown in Table 5, five of the top 10 authors in terms of publications are from the United States. The most highly cited author is McClements, David Julian, who is from the United States, with 2,089 citations. This author has a relatively small number of 26 publications, indicating a high quality of publications, which is favorable for scholars in this field to refer to and learn from.

**Table 5:** Top 10 authors in terms of number of publications.

Rank	Author	Publications	Affiliation	Country	Total citations	Average citations
1	Dewey, Kathryn G	64	UC Davis	United States	1,354	21.2
2	Ashorn, Per	45	Tampere University	Finland	1,032	28.9
3	Ashorn, Ulla	33	Tampere University	Finland	646	19.6
4	Maleta, Kenneth	32	University of Malawi College of Medicine	Malawi	608	19.0
5	Adu-Afarwuah, Seth	31	University of Ghana	Ghana	600	19.4
6	McClements, David Julian	26	University of Massachusetts Amherst	United States	2,089	80.3
7	Arnold, Charles D	22	UC Davis	United States	545	24.8
7	Vosti, Stephen A	22	UC Davis	United States	274	12.5
8	Okronipa, Harriet	20	Oklahoma State University Stillwater	United States	416	20.8
8	Lartey, Anna	20	University of Ghana	Ghana	664	33.2

### 3.6 Analysis of research hotspots

#### 3.6.1 Keyword co-occurrence analysis

**Figure 8:**
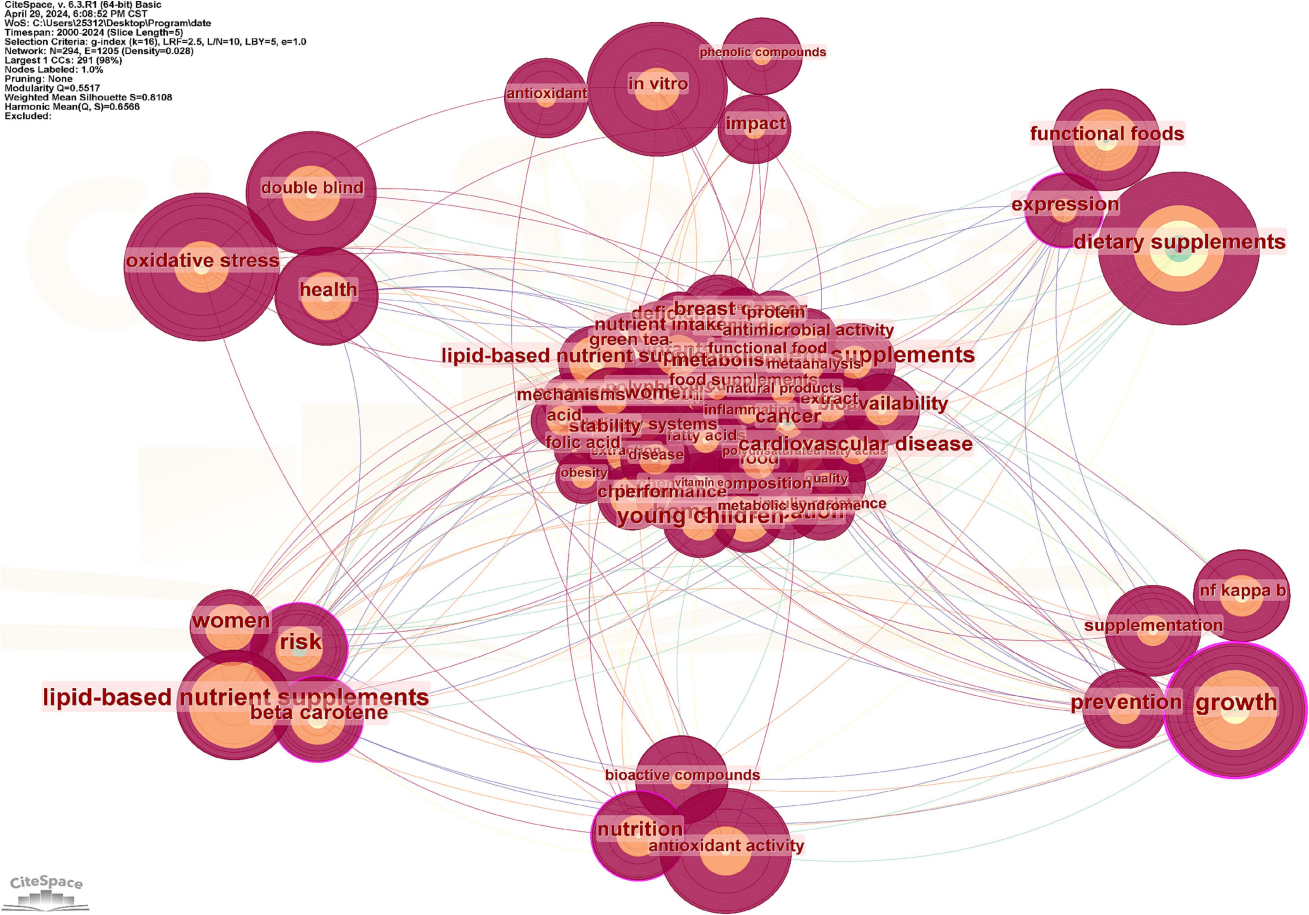
Keyword co-occurrence mapping. The size of nodes is positively correlated with the co-occurrence frequency of keywords, meaning that higher frequency results in larger nodes. The lines represent the co-occurrence relationships between keywords, indicating that these keywords have appeared together in the same literature. Purple circles denote nodes with a centrality greater than 1, and the thickness of these purple circles is proportional to their centrality level; the higher the centrality, the thicker the circle.

There are 294 nodes and 1,205 connecting lines between the keywords, with a grid density of 0.028 (Figure 8). The most frequent keywords were “dietary supplements,” “oxidative stress,” “*in vitro*,” “growth,” “antioxidant activity,” and so on. Among the top 10 keywords in terms of repetition frequency (Table 6), two had a centrality over 0.1, namely “growth” (0.22) and “risk” (0.17). That is, both caused a great sensation in that year and were hot topics of research.

**Table 6:** Keyword co-occurrence frequency.

Rank	Keyword	Occurrences	Centrality
1	Dietary supplements	121	0.07
2	Oxidative stress	117	0.09
3	*In vitro*	103	0.04
4	Growth	100	0.22
5	Antioxidant activity	95	0.07
6	Double-blind	90	0.04
7	Lipid-based nutrient supplements	72	0.06
8	Functional foods	68	0.08
9	Health	61	0.05
10	Risk	51	0.17

#### 3.6.2 Keyword highlighting analysis

**Figure 9:**
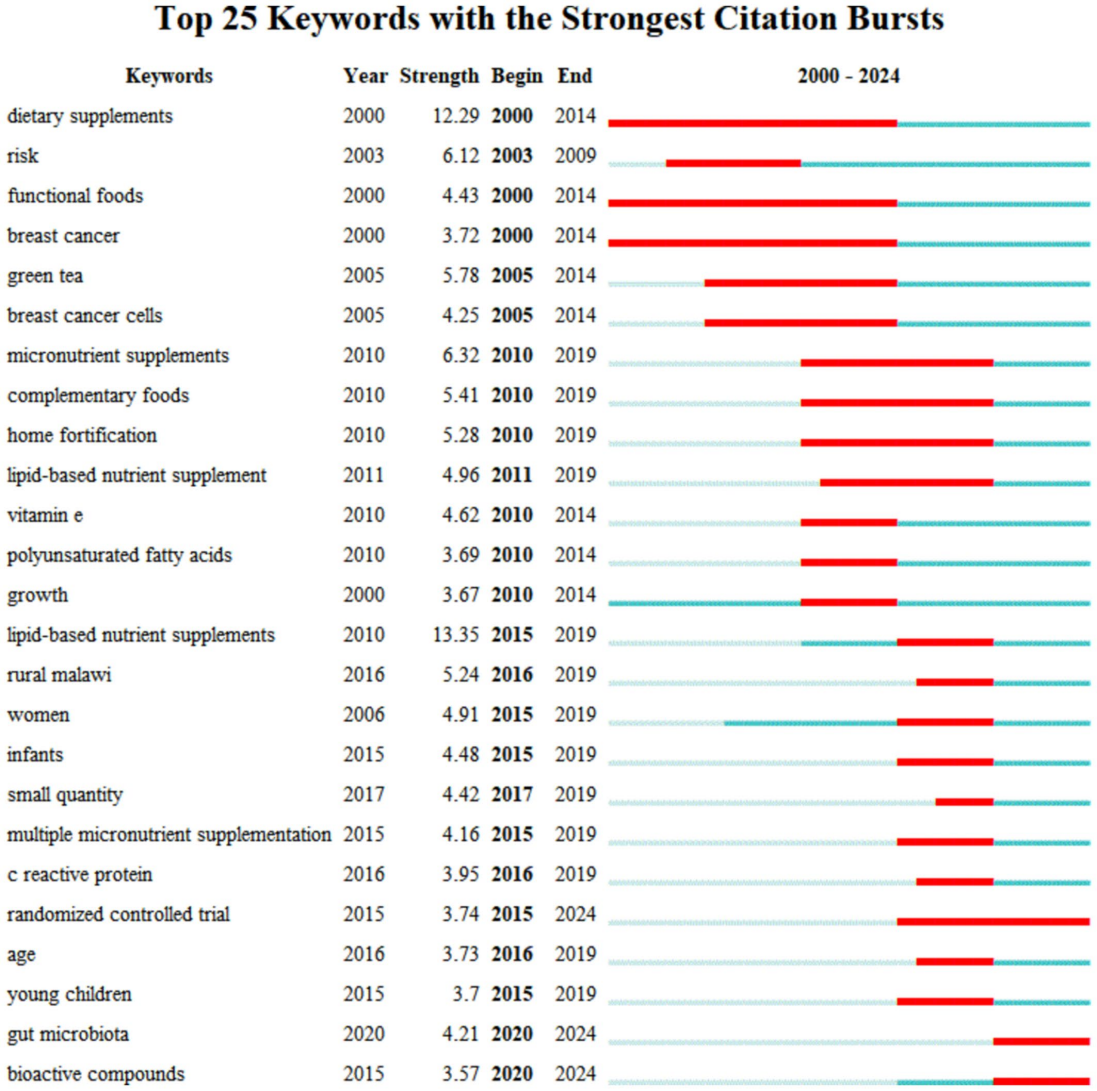
Keyword highlighting. “Strength” indicates the burst intensity of a keyword, with the intensity being directly proportional to its influence. Red lines represent a surge in the usage frequency of a keyword during a certain period, while dark blue lines indicate the opposite trend. The light blue line indicates that research related to this keyword has not yet commenced during that period.

In Figure 9, “strength” represents the burst intensity of keywords, where a higher intensity indicates a deeper impact. The Begin and End points, corresponding to the red line, signify the start and end times of the popularity (37). The length of the red line indicates the duration of the popularity. The dark blue line represents that the topic has been mentioned less frequently by researchers and has not yet formed a research hotspot. The light blue line indicates that no scholars have yet been involved in the research on this topic, and it is still in its nascent stage (38). Keyword highlighting (Figure 9) shows that research on dietary-type supplements, functional foods, and the use of supplements in breast cancer began in 2000 and ended in 2014 as the longest-running hot topic in the field. Lipid nutritional supplement research initially appeared in 2011, with a surge in heat from 2011 to 2019, highlighting a higher intensity. Women and infants became the main subjects of research around 2015. From 2015 to 2024, the randomized controlled trial emerged as the most popular experimental method. Between 2020 and 2024, gut microbiota, bioactive compounds, and randomized controlled trials have seen the highest research heat and are likely to become future trends in development.

#### 3.6.3 Keyword clustering analysis

**Figure 10:**
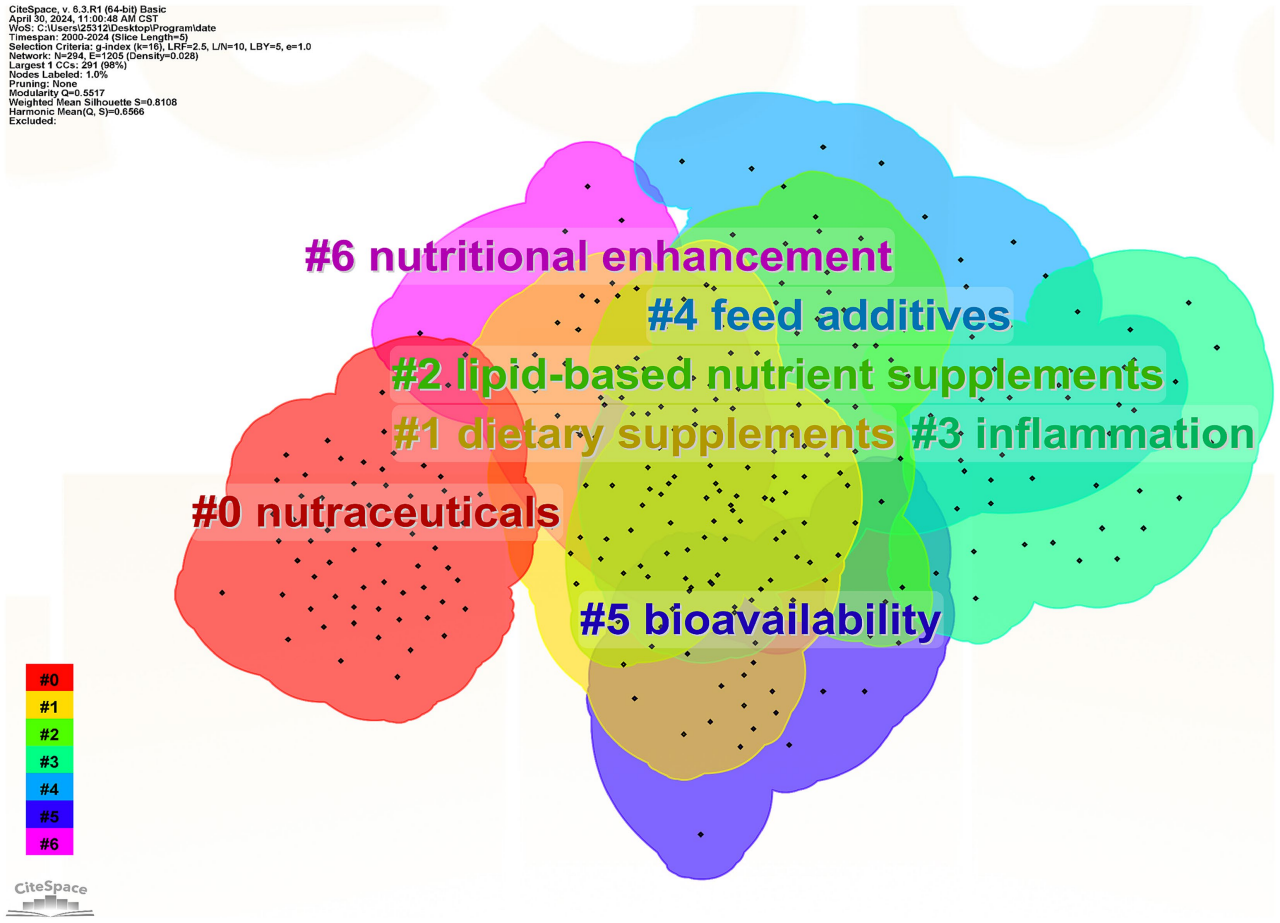
Keyword clustering mapping. Different clusters represent different topics, with nodes of the same color located within the same cluster indicating that they share the same topic. A smaller cluster label number signifies a cluster that contains more keywords, whereas a larger number indicates the opposite.

**Figure 11:**
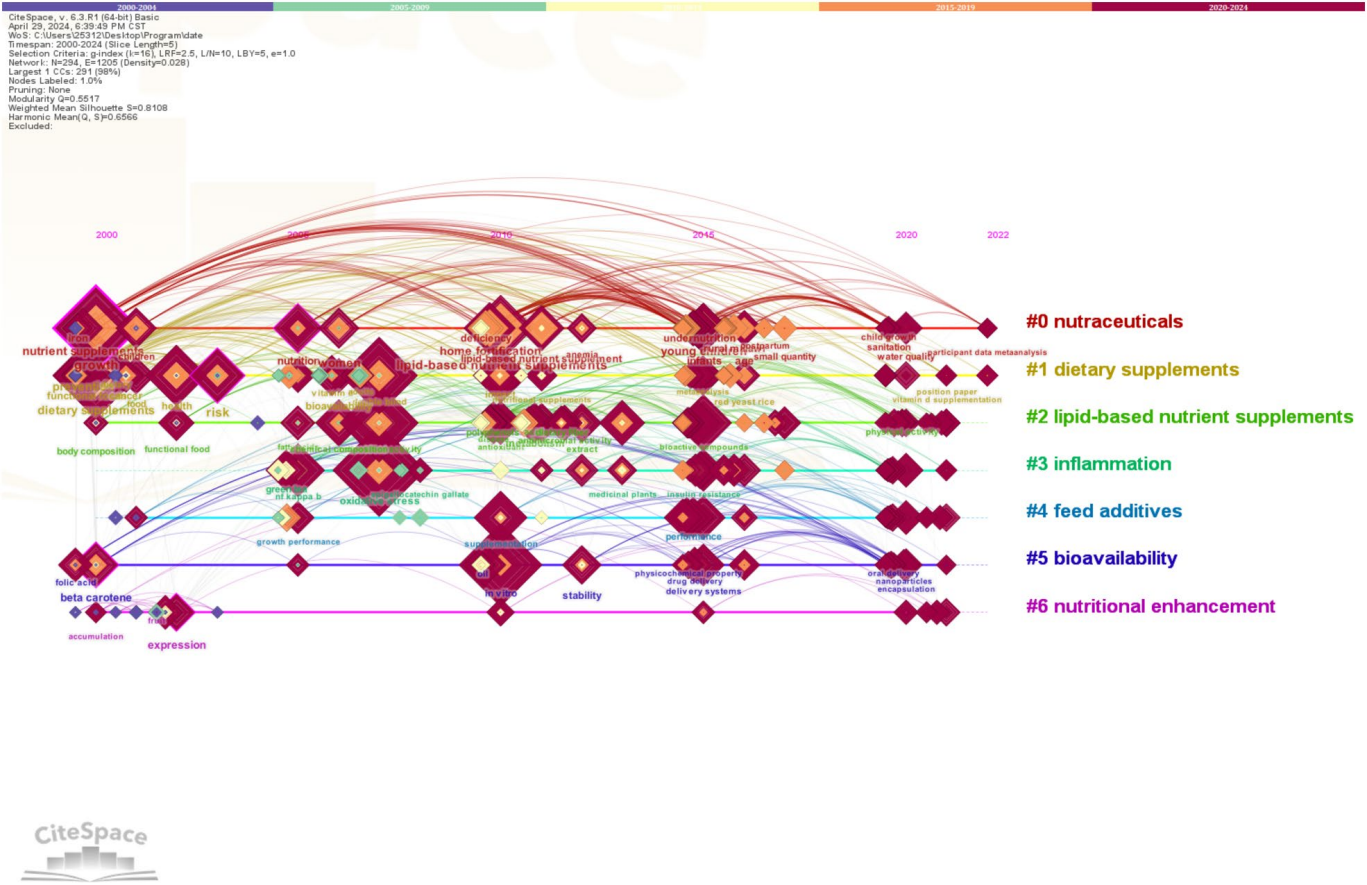
Keyword time zone mapping. Each horizontal line represents a cluster, and the square nodes on the line indicate keywords with higher frequencies of occurrence. The timeline is located at the top of the graph, and the year corresponding to each node represents the time of publication. The lines connecting the nodes reflect their co-occurrence relationships.

Keyword clustering can better represent the research hotspots in the field. In the clustering map, Q represents the module value, which indicates the structure of the clusters; S represents the average profile value, which indicates the clustering efficiency and rationality. Generally speaking, the Q value in the interval [0, 1], Q >0.3, means that the structure of the delineated associations is significant; when the S value is 0.7, the clustering is efficient and convincing, and if it is above 0.5, the clustering is generally regarded as reasonable (39). In this study, the Q value is 0.5517, which indicates that the clustering structure is quite significant, and the S value is 0.8108, which indicates that the clustering results are reasonable, efficient, and convincing (Figure 10). The larger clusters were #0 nutraceuticals, #1 dietary supplements, #2 lipid-based nutrient supplements, #3 inflammation, #4 feed additives, #5 bioavailability, and #6 nutritional enhancement. Figure 11 demonstrates the evolution of keyword research hotspots from 2000 to 2024, in which the high-frequency keywords are primarily “nutraceuticals,” “dietary supplements,” “lipid-based nutrient supplements,” “inflammation,” “feed additives,” “bioavailability,” and “nutritional enhancement.” lipid-based NS and dietary supplementation have consistently informed research in the field. Developmental and dietary supplementation research was most popular around 2000; studies of oxidative stress, antioxidant sensitivity, and double-blind trials were hot topics at the time around 2007. Lipid-based NS and *in vitro* types of supplements gradually came to prominence in 2011. The number of hotspots saw a significant increase between 2015 and 2020, possibly due to technological advancements during that period.

### 3.7 Types functions and effects of nutritional supplements

Table 7 presents the types of supplements (2), the categories of nutrients, and their roles in various diseases. Table 7 shows four significant categories of supplements: amino acids, fatty acids, minerals, and vitamins. Amino acid supplements primarily function to provide additional amino acids, promote protein synthesis, maintain normal bodily functions, enhance immunity, promote muscle growth and recovery, and help regulate nerve function and improve sleep. Fatty acid supplements improve cardiovascular and cerebrovascular health, regulate the immune system, and promote brain function. Mineral supplements primarily maintain bone health, metabolic balance, and the normal functioning of various physiological processes. Vitamin supplements mainly strengthen the immune system, support metabolic processes, and keep the normal functioning of organs such as the skin and eyes. Table 8 demonstrates the impact of NS deficiencies on patient populations in the top 10 countries with the highest publication volume. Vitamin supplements are frequently mentioned in the United States, China, the United Kingdom, Canada, Brazil, and Finland. Cancer has the widest variety of NS types. ONS is used in multiple countries, with different ONS containing various nutrient types corresponding to other diseases. For example, in Italy, high-protein, high-fat ONS are used for individuals with head and neck cancer. ONS containing choline phosphate and docosahexaenoic acid are used for patients undergoing hemodialysis in Spain.

**Table 7:** Types and functions of nutritional supplements.

Nutritional supplements types	Classification	Nutrient	Function
Amino acid	Essential amino acids (114)	Histidine, isoleucine, leucine, lysine, methionine, phenylalanine, threonine, tryptophan, valine	Essential amino acids are primarily responsible for stimulating muscle protein synthesis (114); they have the potential to improve energy levels, mood, and sleep quality (tryptophan, phenylalanine) (115, 116); and they can inhibit the progression of neurodegenerative processes (methionine) (117).
	Non-essential amino acids (118)	Glutamine	Oral glutamine supplements are beneficial for patients undergoing surgery, trauma, transplantation, cancer treatment, wound healing, critically ill newborns, Individuals living with HIV, and endurance athletes (119–122).
	Branched-chain amino acids (123)	Leucine, valine, isoleucine	Preoperative oral administration of branched-chain amino acids can reduce the incidence of post-transplant bacteremia and sepsis in patients who have undergone liver transplantation; it can also promote the phagocytic function of neutrophils and cytotoxic lymphocytes in patients with cirrhosis and reduce mortality (123).
Fatty acids	Polyunsaturated fatty acids (124)	Omega-3, Omega-6	Reduce the risk of cardiovascular disease and metabolic syndrome, prevent or delay cognitive decline and dementia, decrease bone mineral loss, and prevent cancer (124, 125).
	Monounsaturated fatty acids (126)	Omega-7, Omega-9	Reduce blood pressure (127), and improve insulin sensitivity (126).
Minerals	Macro-minerals (2)	Sodium, potassium, calcium, magnesium, chloride, sulfur	Calcium and magnesium are the most common types of these supplements. Magnesium supplementation can reduce the risk of stroke, while potassium and calcium supplementation have been associated with a decreased incidence of stroke in women (128); magnesium supplements can improve insulin sensitivity, and calcium supplements can reduce the risk of atherosclerosis, while also being used to treat osteoporosis (129, 130).
	Trace elements (2)	Iron, manganese, copper, iodine, zinc, cobalt, fluoride, selenium	Iron and zinc are common types of supplements for children. Iron supplementation can treat anemia, while zinc supplementation can treat diarrhea, reduce the incidence of respiratory infections and overall mortality, and benefit children’s growth and weight gain (131, 132).
Vitamin	Water-soluble vitamins (133)	B1 (thiamine), B2 (riboflavin), B3 (niacin), B5 (pantothenic acid), B6 (pyridoxine), B8 (biotin), B9 (folate/folic acid), B12 (cobalamin), and C (ascorbic acid)	Vitamin C supplementation has anti-inflammatory effects and is beneficial for the prevention and treatment of headaches in patients. The use of B2, B6, B9, and B12 significantly reduces the severity of headaches and the associated disability rate (134). B vitamins can be taken as a combination supplement or individually and are beneficial for bone health (133).
	Fat-soluble vitamins (133, 135)	A (retinoic acid), D (calciferol), E (tocopherol), and K (menaquinone)	Vitamin D and vitamin E are the most common supplements. Vitamin D can inhibit pro-inflammatory cytokines and improve insulin resistance, while vitamin E can improve oxidative stress and inflammatory conditions (136). Vitamin A helps reduce the risk of all-cause mortality and measles incidence (135). Vitamin K can prevent clinical fractures (133).

**Table 8:** Impact of nutritional supplement deficiency on the sick population in different countries.

Country	Deficiency of nutritional supplements	Impact
United States	Antioxidant (106)	High concentrations of reactive oxygen species increase the survival rate of cancer cells in individuals with cancer.
	Multivitamin/multimineral supplement (137)	Inadequate intake of magnesium and vitamins A, C, D, E can contribute to an increased incidence of various diseases (including neural tube defects, osteoporosis, immune dysfunction, cognitive impairment, chronic conditions, eye diseases, hypertension, coronary heart disease, and stroke)
Italy	Oral nutritional supplements (high protein, fatty acids) (138)	Individuals with head and neck cancer often suffer from malnutrition, weight loss, and low body mass index due to chewing and swallowing difficulties. Before or during radiotherapy, they experience reduced protein and calorie intake, leading to poor prognosis, decreased quality of life, increased treatment toxicity, and the development of severe mucositis.
	Red yeast rice (monacolin) and/or plant sterols (139)	Individuals with hypercholesterolemia have elevated levels of low-density lipoprotein cholesterol, which increases the risk of cardiovascular mortality and morbidity.
India	Liver-protecting supplement (40)	Individuals with liver disease may experience increased fat levels, cirrhosis, and low hydration levels.
	High-protein supplement (140)	Protein-energy malnutrition in individuals undergoing hemodialysis increases morbidity and mortality.
China	Selenium supplement (141)	Selenium deficiency can lead to Kashin–Beck disease.
	Multivitamin/multimineral supplement (142)	Individuals with Type 2 diabetes experience weight gain and obesity.
England	Oral nutritional supplement (calories and protein) (143)	Malnutrition in individuals undergoing hemodialysis can lead to increased mortality, morbidity, decreased quality of life, and an elevated risk of hospitalization. For example, anorexia caused by uremic toxins, inadequate oral protein intake, protein loss during dialysis, and catabolism can all lead to malnutrition.
	Eye nutrition supplement (fatty acids, antioxidant vitamins, minerals) (144)	Age-related macular degeneration (AMD) can lead to rapid vision loss, debility, and an increased risk of depression.
Spain	Oligomer-based high-protein normal-calorie and immune-nutritional supplement (145)	Malnutrition in individuals with colorectal cancer increases the risk of infections and postoperative complications.
	Oral nutritional supplement (citicoline and docosahexaenoic acid) (146)	Individuals with glaucoma experience worsening nerve damage, and increased intraocular pressure, which ultimately may lead to blindness.
Australia	Protein nutritional supplement (147)	Malnutrition can impair wound healing in individuals with chronic wounds, including pressure ulcers and surgical wounds.
	Energy-dense oral nutritional supplement (fatty acids, protein) (148)	Malnutrition in individuals with lung and pancreatic cancer can lead to weight loss, increased risk of complications, decreased response and tolerance to treatment, reduced quality of life, and decreased survival rates.
Canada	Multivitamin/multimineral supplement (149)	Consumption of beef, pork, or lamb as main dishes and processed meats increases the risk of kidney cancer. Among men who smoke or are overweight, there is a positive correlation between red meat and processed meat consumption and kidney cancer.
	Oral nutritional supplements (protein-energy and protein-only types) (150)	Malnutrition and protein-energy wasting in individuals with non-dialysis chronic kidney disease increase the risks of frailty, cardiovascular disease, infectious complications, and mortality.
Brazil	Vitamin D combined with sulforaphane (151)	Insufficient Vitamin D Levels in individuals with prostate cancer increase the risk of disease advancement.
	Glutamine (152)	Low-grade chronic systemic inflammation in the elderly increases the risk of insulin resistance, cardiovascular disease, type 2 diabetes, and neurodegenerative diseases.
Finland	Vitamin and calcium supplements (153, 154)	Individuals who have undergone sleeve gastrectomy often experience nutritional deficiencies (such as vitamin D and calcium) postoperatively, which can subsequently lead to secondary hyperparathyroidism and osteoporosis.
	Oral nutritional supplement (rich in protein and energy) (155)	Malnutrition in individuals with colon cancer can lead to increased postoperative morbidity and mortality.

## 4 Discussion

Nutritional supplements are used as products intended to provide essential nutrients that may be lacking in sufficient quantities in the human diet (40). However, a significant number of patients do not know what an NS is and its function and consider it a drug. This may mislead patients into believing that supplements are as safe and effective as drugs and that drugs are studied in the same way as NS (41). The current use of NS is not standardized, and a large percentage of people do not consider the opinions of healthcare professionals who believe that what is naturally made is safe and effective. For example, in the United States, less than a quarter of NS is recommended by a physician or medical professional (42). A non-randomized observational trial of postmenopausal women showed a significant 6% increase in mortality in women taking MVM (43). In a Swedish population-based cohort study of men aged 45–79 years, the use of high-dose vitamin C or E supplements was associated with an increased incidence of age-related cataracts. This shows that although NS has an important role to play in disease prevention or recovery, it should be tailored for consumers according to their age, gender, family history/risk factors, and stage of life (including reproductive age and old age) (16). Therefore, this paper compiles the research hotspots and dynamics in this field, which can help people quickly understand the relevant general knowledge and knowledge in this field and promote the use of NS for sick people to be more rationalized and standardized.

The trend of the annual number of papers can reflect the development speed and research heat of this research field in a certain period. Through the statistics of the annual number of papers, we can grasp the development stage and change trend (44). The research on NS for diseased populations in the past 20 years has been divided into three main phases. The number of papers issued from 2000 to 2008 was at a steady stage, and the research was primarily carried out in the direction of functional foods, antioxidants, dietary supplements, anthocyanins, and so on. Mazza, G was the more influential author in this period, who was primarily engaged in the research on the extraction of nutrients from plants for the prevention and treatment of diseases. Lacquer tree includes more than 250 species of flowering plants, and its extracts are known for their antibacterial, antifungal, and antiviral activities (45), which can be used for the treatment of bacterial diseases such as syphilis, gonorrhea, dysentery, and gangrene (46). This author optimized the extraction of phenolic compounds from berries, which are known to reduce coronary heart disease, prevent many types of cancers, and treat urinary tract disorders, as well as anti-inflammatory and antioxidant activities. Anthocyanins are the main phenolic constituents of berries and are twice as effective as commercially available antioxidants such as BHA and *α*-tocopherol (47). The 2009–2017 period has been characterized by a continuous growth in the number of publications, which is the accumulation phase of the field. This period was dominated by studies on oxidative stress, NS during development, and lipid NS. The results showed that lipid NS (LNS) are usually made from vegetable oils, milk powder, peanut butter, sugar, and a variety of micronutrients (48). There are various formulations of LNS, such as small-quantity LNS (SQ-LNS), used for preventing malnutrition and promoting growth and development (49). Studies during this period found that providing LNS-containing milk might promote growth from infancy to childhood (50). After 2018, the number of publications increased dramatically, and the field entered a phase of rapid growth. Studies on oxidative stress, *in vitro* nutritional supplementation, and antioxidant activity were mainly conducted during this period. Studies have shown that polyphenols, which are low molecular weight compounds present in seeds, flowers, and fruits, have antioxidant properties (51–53). Phenolic compounds are present in red wine, and proper daily consumption of red wine can reduce oxidative stress and inflammation, which can be beneficial in preventing coronary heart disease (54, 55). In the last two decades of research in this field, experiments and tests are often conducted using double-blind experiments and RCTs. Double-blind trials are designed to reduce the influence of potential bias on peer review in the pursuit of objectivity and fairness (56). RCTs are a major factor in the rapid development of medical science, marking the development and refinement of clinical research methods (57). This methodology plays a key role in modern clinical research and is important for the level of evidence in evidence-based medicine (58).

Nutrients ranks first in terms of the number of articles published and the frequency of citations, with a total of 64 articles published and 1,965 citations. It shows that this journal has a large influence in the field, and the quality of articles is high, with strong academic value and status. In the past two decades, the number of articles published by this journal has been on a steep rise; the number of articles published in 2019 surged, and the overall growth trend remained unabated until 2023. The journal covers a wide area of NS research, focusing on oxidative stress, dietary supplements, inflammation, chronic kidney disease, muscle mass, and vitamin D. The results show that adequate nutritional intake is essential for developing a healthy diet, addressing surgical stress, and mitigating the loss of muscle mass, strength, and function. Emphasizing protein intake during surgery, especially after surgery, can reduce muscle catabolism, resulting in loss of function (59). Egg white contains all the essential amino acids needed and has many beneficial effects on the body, and supplementation of an egg white-formulated diet is expected to improve nutritional status and increase serum albumin levels (60). Oxidative stress has been found to activate inflammatory pathways and enhance oxidative stress, which can accelerate the progression of metabolic diseases (59, 60). However, antioxidants such as polyphenols and resveratrol, along with vitamins C and E, can alleviate oxidative stress in metabolic disorders by enhancing the body’s natural antioxidant defenses and reducing reactive carbon production (61, 62). The journal also conducted a study on whether biological supplements are effective in improving the prognosis of People with kidney disease. Chronic kidney disease is associated with high morbidity and mortality, and its global prevalence is estimated to be around 13.4%. Previous meta-analyses have found that ONS improves the nutritional status of People undergoing long-term dialysis and may reduce complications (63, 64). A related study found a significant improvement in residual renal function in People with Parkinson’s disease 6 months after intervening with probiotic supplementation (65). Vitamins are active hormones in the liver and kidneys (66), and they are classical regulators of calcium and bone metabolism. Among its classical endocrine functions, vitamin D regulates mineral metabolism (67). Combining vitamin D supplements with protein supplements and exercise has been shown to increase patients’ grip strength and has also shown a tendency to increase muscle mass (68). Vitamin D (alone or in combination therapy) is effective in enhancing muscle strength and function in older adults (67, 68). “Methylsulfonylmethane (MSM): Applications and Safety of a Novel Dietary Supplement” was the most cited article in the journal. The article noted that MSM has become a popular dietary supplement that improves a variety of health-specific outcome metrics, including inflammation, joint/muscle pain, oxidative stress, and antioxidant capacity (69).

“Phytochemicals: nutraceuticals and human health,” the most cited article, focuses on research conducted on whether phytoconstituents can be incorporated as NS based on their ability to protect and promote health. Studies have shown three main groups of phytochemicals, namely terpenoids, phenolic metabolites, and alkaloids, and other nitrogenous plant constituents (70). The “new” nutraceuticals of plant origin may evolve into an important aspect of disease-preventive food composition (71).

In the global field of nutritional supplementation for sick populations, the top institutions are conducting research in the following directions: (1) The UC System in the United States focuses on lipid supplements and developmental research, primarily on children and women. The results show that lipid supplements provide micronutrients and key macronutrients (including fatty acids) (72) and can enhance the amount of micronutrients needed during pregnancy and lactation. Lipid supplementation during pregnancy (LNS-PL) also reduced neonatal growth retardation, wasting, and microcephaly in the study population (73). (2) The UC Davis and the UC System are similarly oriented in terms of the populations that they address, which are mostly women and children. We found that the top highly cited article was also from UC Davis. The study showed that prenatal and postnatal provision of SQ-LNS may be effective in improving linear growth in children (74). (3) The University of Tampere, Finland, focuses on LNS, development, and malnutrition and caters to a population of infants and children. The results showed that in women, prenatal supplementation with SQ-LNS promoted an increase in infant birth weight and height, as well as a decrease in the prevalence of Small for Gestational Age and neonatal growth retardation (75). The addition of SQ-LNS to the complementary diet of children aged 6–23 months may improve growth and anemia and may be more effective than other alternatives in improving growth (76). (4) The University Hospital of Tampere from Finland is like the University of Tampere study in that both have conducted research on lipid supplements, development, and so on and have dealt with infants and children. They work closely together, and the articles are closely linked. (5) The University of Malawi from Malawi focuses on lipid supplements, development, and malnutrition. Most of the populations targeted were infants, children, and women. Results showed that 12 months of dietary supplementation with LNS promoted linear growth and reduced the incidence of severe growth retardation in rural Malawian infants and young children (77). Another study indicated that the intake of LNS containing milk and soy had little impact on the participants’ linear growth and incidence of severe growth retardation, but certain antinutrients such as enzyme inhibitors, phytates, and lectins could potentially exert negative effects on linear growth (78).

The research and views of the team of highly productive and highly cited authors in the field of NS for diseased populations are shown as follows: (1) The Dewey, KG team from the United States primarily engages in research on LNS. The target population is primarily infants, women, and children. The results showed that SQ-LNS did not reduce depressive symptoms in Ghanaian women at 6 months postpartum (79) but increased plasma selenium concentrations in pregnant women (80). Nutrition-focused healthcare quality improvement reduces healthcare costs for patients and provides a high-value medication guideline for malnourished hospitalized populations (81). (2) The team of Ashorn, Per from Finland found that the provision of SQ-LNS reduced the prevalence of anemia (Hb < 110 g/L) by 16% (a relative reduction) and could significantly reduce childhood anemia, iron deficiency, and iron deficiency anemia (82). (3) The team of Ashorn, Ulla from Finland found that SQ-LNS supplementation in children aged 6–18 months led to earlier independent walking and waving goodbye (83). (4) The team of Maleta, K from Malawi found that the provision of iron-containing LNS to infants and young children for 12 months did not increase the prevalence of infectious diseases (84). (5) The team of Adu-AFNS from Ghana found that the provision of SQ-LNS to infants and young children did not increase the prevalence of infectious diseases (85). Adu-Afarwuah et al. (86) from Ghana found that antenatal supplementation with LNS improved fetal growth in vulnerable women in Ghana, especially primiparous women.

In the field of NS, dietary and lipid supplements are used most frequently and belong to the core types of the field. They are primarily used in People with cancer, patients with malnutrition, women, and children (87–89). Malnutrition may be due to personal, environmental, and food factors, such as individuals who are more sensitive to cholecystokinin (which suppresses appetite) experiencing faster satiety (90). The rate of glucose release in response to food may also affect appetite (91). The probability of being underweight is as high as 12.5% among community-dwelling People with chronic illnesses (92). The average prevalence of underweight in patients admitted to hospitals has been estimated to be about 18% (range 5–37.5%) (93). On average, patients with nutritional risk have a 19% higher hospitalization cost than at-risk patients with similar diagnoses (94). Providing nutritional support to patients with malnutrition can reduce the rate of postoperative complications by 10%, as well as save healthcare costs and reduce hospitalization time (95). Oral nutritional interventions given to people with cancer who are malnourished or at nutritional risk can be effective in increasing nutritional intake and improving quality of life (96). Gastrointestinal people with cancer and people without cancer both prefer the taste of fresh milk-based supplements (97). Dietary supplements, functional foods, and breast cancer were highlighted with higher intensity around 2000–2015, which was the dominant direction of research at that time. The results showed that patients with and survivors of breast cancer often use complementary and integrative therapies (98) and that breast cancer treatment could be improved by complementary dietary supplements (99). Studies during this period found that dietary bioactive compounds from different functional foods, including herbs and nutraceuticals (ginseng, ginkgo, nuts, grains, tomatoes, soy phytoestrogens, curcumin, melatonin, polyphenols, antioxidant vitamins, carnitine, carnosine, myostatin, ubiquinone, etc.) could improve health and prevent chronic diseases associated with aging, such as promoting antioxidant activity, mitochondrial stabilization function, metal chelating activity, inhibition of apoptosis in important cells and induction of apoptosis in cancer cells (100). In 2015, the field gradually shifted to research on multiple-micronutrient supplementation and lipid nutritional supplementation. Women, infants, and children became the focus of attention. It has been found that malnutrition (nutritional deficiency or obesity) in pregnant women can cause or exacerbate a large number of problems, such as anemia, maternal bleeding, insulin resistance, and hypertensive disorders, and also affects the normal development of the fetus during pregnancy (101). Supplementation with appropriate amounts of multiple micronutrients is beneficial in reducing the number of less-than-gestational-age children and maternal anemia (102). In 2020, the research direction gradually shifted toward the study of bioactive compounds and microbiota. The human gut contains a wide range of bacteria that have important metabolic and immune functions, and the gut microbiota produces metabolic activities that contribute to the digestion of dietary compounds, the rescue of energy, the provision of micronutrients, and the transformation of exogenous substances (103). Relevant researchers have found that healthy dietary patterns and supplements with bioactive compounds can be used as simple and easy interventions to prevent, mitigate, or cure clinical diseases, especially degenerative and chronic diseases (104).

In summary, the use of NS in diseased populations is very valuable. If used in the right way, type, and dosage, they will have unique benefits for prevention and rehabilitation and can reduce home healthcare costs, as well as the number of hospitalizations and days patients are hospitalized. However, the research on NS in the sick population is still one-sided; for example, the survey experiments are not sufficient to support the assessment of the effect of NS on the overall patient population. Risk assessment of the use of NS, its abuse in some populations without the guidance of healthcare professionals, clinical trials, and its effects on preoperative and postoperative periods in different populations are all issues that need to be improved in this field. Therefore, research on NS for sick people should continue, and the research efforts and regulations should be increased to solve and improve existing problems and promote the rational use of NS to help human society.

## 5 Limitations

In this paper, a bibliometric analysis of the field of NS for diseased people was conducted with the help of Citespace and VOSviewer. The use of visual mapping reveals more intuitively the trends and patterns in the field, which in turn derives a series of new findings and new interpretations. This study only selected the literature in the WOSCC database and did not include books and other databases; thus, the coverage of the selection is not sufficient to support the overview of the entire field. Furthermore, this study has certain limitations in selecting patient data between 2000 and 2024. Due to the scarcity of relevant literature before 2000, the study could not include data from that period for analysis, which may result in inadequate coverage of earlier research trends. However, since 2000, the number of related papers has increased significantly, providing abundant data sources and research support for this study. Nevertheless, this selection may still impact the historical continuity of the research conclusions. This paper focuses on the number of publications, journals, authors, institutions, keywords, and countries, aiming to provide a macro-analysis of the field; thus, some details are not reflected in the study, leading to a limited analysis of the literature. In this study, the search period was set to January 2000–April 2024, and articles after this period were excluded from the analysis list.

## 6 Conclusion

Findings show that the field of NS for sick populations has witnessed an overall rapid growth in the number of publications, which is divided into three phases. Subsequently, 2000–2008 was the exploratory phase, 2009–2017 was the sustained development phase, and 2018 to date is in the rapid development phase. The United States is the country with the highest number of publications in the world. The UC System in the United States is ranked as the leading institution in terms of the number of articles issued. Nutrients is the most published journal. Dewey, KG, is the most published author. The top highly cited article in the field is “Phytochemicals: nutraceuticals and human health.” Dietary supplementation, oxidative stress, *in vitro* injections, development, antioxidant activity, double-blind trials, LNS, functional foods, health of diseased populations, and risk of NS use are the top research topics in this field. Women and infants are the primary research subjects. Dietary-type supplements, functional foods, and supplement use in patients with breast cancer are the longest-running studies in the field. Different types of supplements each possess unique benefits, and should be chosen according to the type of disease to ensure they contain the corresponding nutrients. Vitamin supplements are widely mentioned among patient populations across the globe. Future trends in this area may focus on the application of nutritional supplements in gut microbiota and bioactive compounds.

## Data Availability

The original contributions presented in the study are included in the article/supplementary material, further inquiries can be directed to the corresponding author.
